# A Co-extrusion Additive Manufacturing Process with Mixer Nozzle to Dynamically Control Blowing Agent Content and Print Functionally Graded Foams

**DOI:** 10.1021/acsaenm.4c00764

**Published:** 2025-03-14

**Authors:** Karun Kalia, David Kazmer, Amir Ameli

**Affiliations:** Department of Plastics Engineering, University of Massachusetts Lowell, 1 University Ave., Lowell, Massachusetts 01854, United States

**Keywords:** Additive Manufacturing, 3D Printing, Co-extrusion, Foaming, Functionally Graded Foams, Mixing, Mechanical Performance

## Abstract

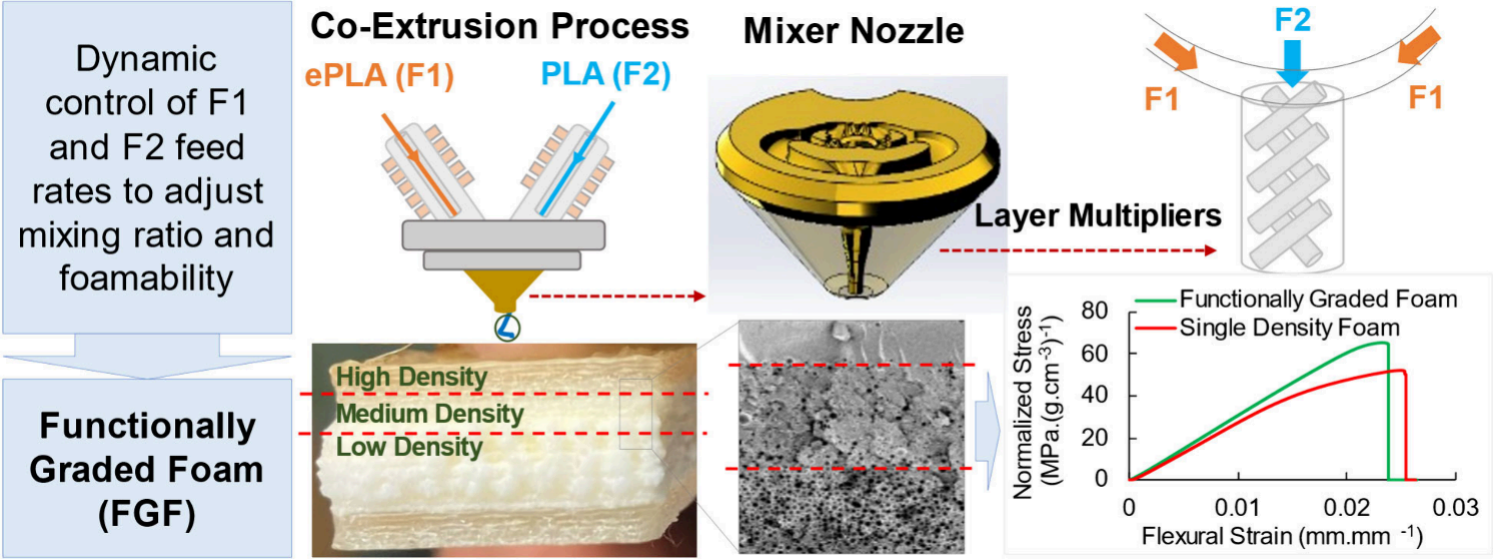

A unique approach to 3D-print functionally graded foams (FGFs) via dynamic control of the blowing agent content is demonstrated. The approach utilizes a co-extrusion additive manufacturing process equipped with a static mixer nozzle (SMN) and thermally expandable microspheres (TEMs) as the foaming agent. The nozzle consists of two flow paths, one longer than the other, to facilitate the feeding of two different filaments. It is also equipped with layer multiplying elements (LME) for the mixing of the incoming melt streams. The first incoming filament was the expandable polylactide acid loaded with 8.0 wt % TEM (ePLA) to be mixed with the second filament made of neat PLA. The mixing of the two filaments at various ratios was successfully achieved, resulting in foams with uniform cellular morphologies at various densities. The choice of flow path also had a significant effect on the foam density. When ePLA was fed through the longer flow path, a greater degree of foaming was obtained due to a longer residence time. The FGF flexural samples, printed through this method, demonstrated a superior mechanical performance compared to their single density foam and solid unfoamed counterparts. The results reveal that this approach of foam additive manufacturing process provides a capable method to manufacture complex and functionally graded structures with programmable density profiles with specific gravities varying between 0.43 and 1.21 g cm^–3^ on demand.

## 1 Introduction

Functionally graded materials (FGMs) comprise of one or more materials that are designed and structured in a certain fashion to provide spatially variable attributes.^1,2^ In the context of polymeric materials, manufacturing such structures with traditional injection molding and extrusion processes is a challenging task due to tooling complexities and cost. With the advent of additive manufacturing (AM), multimaterial additive manufacturing (MMAM) processes have emerged as potential solutions, which enable the fabrication of multimaterial structures with complex design geometries and various materials integrated with discrete chemical, thermal, or physical properties.^3^ Several research efforts have focused on the fabrication of FGMs using MMAM processes and argued that MMAM can provide an efficient way to fabricate FGMs by saving material and production cost.^2−4^

Material extrusion (MEX) is a widely used AM process for polymer-based materials and structures. The direct deposition of thermoplastic polymers and composites enables part creation with road-by-road and layer-by-layer material control. There are several processing approaches in MEX AM to make functionally graded materials and structures. One way is to utilize a single nozzle with a single material and obtain functionally graded structures through part design (e.g., variable infill density, variable lattice structure)^2^ or controlling the process conditions.^5,6^ Functionally graded structures have certainly provided improved multifunctional properties, and, when such structural designs are manufactured with the aid of controlled processing conditions, then it provides the possibility to meet the demands of topological optimized structures for a wide range of applications in the field of biomedical implants, energy-absorbing structures, compliant structures,^7^ optoelectronic devices, etc.

Another processing approach is the utilization of multiple materials with single or multiple nozzles.^8,9^ Baca et al. demonstrated the use of multiple nozzles with three different material combinations of acrylonitrile butadiene styrene (ABS), high impact polystyrene (HIPS), and polylactic acid (PLA). They reported poor interfacial adhesion as a significant challenge with this approach. They also examined printing of all three materials using a single nozzle in a sequential manner, which improved the interlayer adhesion due to reduced temperature gradients and print times.^10^ It should be noted that such a setup can perform well only for materials with similar melting temperatures. Previously, attempts have also been made in optimization of tool path navigation for multiple nozzle prints, which helps in reducing the print time and filament usage.^11^

A third processing approach is to use multiple materials/filaments which are simultaneously fed to one exit nozzle, known as the co-extrusion process. In this method, several filaments are fed to the single-nozzle hot end assembly, which are then combined in different configurations including homogeneous mixtures^12,13^ and core/shell structures^14^ at varying ratios before extrusion. Compared to multinozzle MMAM, the co-extrusion approach improves upon the issues of poor interface adhesion between two completely different materials, limited printing resolution, and longer fabrication time.^3^ The single nozzle used in the co-extrusion AM process can also be equipped with an in situ mixer to provide a better blending of the incoming melt streams. Kennedy et al.^12^ designed a dynamic mixer inside a extruder head of a MEX 3D printer. The rotational speed of the mixing element (inside the extruder head) and the input polymer flow rates were tuned simultaneously to investigate the blend morphologies of the thermoplastic polyurethane/poly(lactic acid) (TPU/PLA) and nylon/PLA compositions. Khondoker et al.^13^ showed a custom designed co-extrusion 3D-printer setup with two filament inlets merged to a channel with a static mixing element and single nozzle exit. Their results showed that the intermixed extrudates had reduced delamination issues compared to parts printed with side-by-side deposition of the melts using two separate nozzles. Brackett et al.^15^ implemented a static mixer nozzle (SMN) in the MEX large-scale AM process and used carbon fiber reinforced acrylonitrile butadiene styrene (CF/ABS) and neat ABS filaments to mix them during extrusion. Lahaie et al.^16^ demonstrated various designs of in situ SMN with an objective to improve the dimensional stability of the co-extruded ABS and polycarbonate (PC) components.

One class of FGMs is functionally graded foams (FGFs), which can be made of one or more materials.^17,18^ In FGFs, the primary spatial variable is the density of foam, which can subsequently provide other functionalities such as graded modulus of elasticity and graded strength.^19,20^ In this approach, although multiple materials can be used, they are not necessarily needed. Using only a single material and manipulating the density and cellular morphology provide functionally graded structures. FGFs tend to have enhanced functional properties. For instance, Cui et al. conducted a numerical simulation to demonstrate the energy absorption characteristics of various FGFs.^21^ Mosanenzadeh et al. reported the enhanced acoustic capabilities of bio-based FGFs.^22^ Dileep et al. reported enhancement in the buckling strength of FGFs made of glass microballoons incorporated in a high-density polyethylene (HDPE) matrix.^23^ As previously shown, FGFs made with thermally expandable microspheres loaded in a PLA matrix showed improved energy absorption capabilities in both low velocity impact and quasi-static compression testing.^24^

Compared to single density foams (SDFs),^25,26^ FGFs have shown to provide advantages in terms of high mechanical performance, and there have been several numerical and experimental reports on understanding their mechanical behavior.^19,23,27−30^ Generally, FGFs are made using various manufacturing processes such as batch foaming,^31,32^ extrusion foaming, foam injection molding,^33,34^ and particulate leaching.^22^ However, obtaining a controllable gradient in cellular structure or density using these conventional manufacturing processes is still a challenge. Another method is to adhesively bond several layers of discrete densities^35,36^ at the expense of additional postprocessing and debonding issues during service.

Foam AM has recently been the focus of some studies.^37,38^ Kalia et al. have shown the successful in situ foam 3D printing via the MEX AM process where thermally expandable microspheres (TEMs) were used as the foaming agent together with PLA as the base material.^25,26^ In a recent study by the authors, the 3D printing of FGFs obtained using a single filament through process control was also demonstrated.^24^ Nozzle temperature and flow rate were identified as the key print process parameters, and with a concurrent change in nozzle temperature and flow rate parameters, a maximum density gradient of 0.86 g cm^–3^ was achieved. Recently Epasto et al. mechanically characterized the sandwiched beams printed at various densities via the MEX AM process where the feedstock filament was impregnated with CO_2_ gas as a foaming agent.^14^ In all such approaches, only print process parameters were the variable factors, whereas input material was kept constant. Another innovative approach to print FGFs would be through the dynamic control of the amount of blowing agent, which subsequently controls the degree of foaming and expansion. This approach not only provides an additional degree of freedom in process design but also enables utilization of the maximum expansion capability of the blowing agent (TEMs). This approach has not yet been reported in the literature, and it is the subject of this work.

In this work, to assess the feasibility of in situ control of the blowing agent content and create FGFs therefrom, a co-extrusion printer with a custom feed manifold and built-in static mixer was designed. A commercially available Creality CR-X Pro 3D printer was adapted to incorporate two feeding intakes with a single-nozzle hot-end assembly. A custom hot-end assembly equipped with an SMN was designed, made, and integrated into the printer. Feedstock filaments, one foamable filament (ePLA), and another unfoamable filament (PLA) were made using a single-screw extrusion line. Both filaments were then simultaneously fed to the hot-end assembly with predetermined relative feed rate ratios, mixed inside the SMN, and extruded. The change in the feed rate ratios of the two filaments effectively controlled the mixing ratios of the two filaments and, thus, the blowing agent (TEM) amount in the extruded melt. The effect of the flow paths inside the SMN as well as the effect of the mixing ratios of the two filaments were studied and analyzed. To demonstrate the feasibility and performance, FGFs with controlled cellular morphologies and densities were made using this approach and compared with their SDF and unfoamed counterparts in terms of their flexural behavior.

## 2 Experimental Section

### 2.1 Materials and Filament Fabrication

NatureWorks Ingeo 4043D polylactic acid was used as the polymer matrix and dry mixed with Sekisui Advancell EM501E1 thermally expandable microspheres at 8.0 wt % loading of TEM before feeding to the hopper. The TEM grade of EM501E1 is a masterbatch with 50 wt % polyethylene carrier and 50 wt % TEM and has an initial particle size of 21–31 μm. The TEMs have a start expansion temperature (*T*_start_) and maximum expansion temperature (*T*_max_) of 160–180 °C and 210–230 °C, respectively. Filaments were processed using a Dr. Collin E30P single screw extruder with a screw length to diameter ratio of 25. The screw profile consisted of a Maddock mixer at the end to provide better mixing.^39^

### 2.2 Co-extrusion Printing Process

The Creality CR-X Pro 3D printer was modified with a custom designed hot-end assembly equipped with an in situ static mixer nozzle. Marlin firmware 2.1 on the Creality printer and the G-codes of the print files were also modified with proper extruder commands to activate the concurrent feeding of dual filaments. PLA and ePLA filaments were simultaneously fed through tools 0 and 1, respectively, for the first flow path configuration. The filaments were swapped between tool 0 and tool 1 for the second flow path configuration. More details are provided in Section 3.2.3. Feeding rates and thus the mixing ratios of the two filaments were controlled by using M163 and M164 G-code commands. Table 1 shows the G-code commands used to print only PLA (ePLA:PLA = 0.0:1.0), a mix of ePLA and PLA (for instance, ePLA:PLA = 0.5:0.5), and only ePLA (ePLA:PLA = 1.0:0.0). These commands were added to the original G-code file. M163 is a command to set the ratio of the mixed material, followed by *S*’*n*’ where *n* denotes the tool number and *P*’*n*’ where *n* denotes the feeding ratio from that tool. M164 is a command to store these feed weightings in a virtual extruder, which can have any number ‘*n*’ lower than 12 (limited to firmware version), except 0 and 1 (as S0 and S1 refer to tool 0 and tool 1). For instance, if the commands shown in the second row of Table 1 are added to the G-code, the printer will feed 0.5 from tool 0 and 0.5 from tool 1, resulting in a mix of 50:50. If the filament feed-in *E* value is 2.0 mm, the firmware automatically adjusts the extruder’s stepper motor rpm such that each of tool 0 and 1 simultaneously feeds 1.0 mm of filament, providing the required *E* of 2 mm. This approach dynamically controls the feeding rate of both the filaments, which will set the TEM loading in the SMN and thereby result in different degrees of foaming and densities. Several single density foams, functionally graded foams, and solid PLA flexural samples were then printed and characterized.

**Table 1:** Mixing Commands Needed in the G-Code before the Start of Layer Printing to Print at Various Mixing Ratios of PLA and ePLA

Feeding and mixing ratios	G-code commands
ePLA:PLA = 0.0:1.0	M163 S0 P0.0
	M163 S1 P1.0
	M164 S3
	T3
ePLA:PLA = 0.5:0.5	M163 S0 P0.5
	M163 S1 P0.5
	M164 S5
	T5
ePLA:PLA = 1.0:0.0	M163 S0 P1.0
	M163 S1 P0.0
	M164 S8
	T8

### 2.3 Characterizations

#### 2.3.1 Density and Microscopy

The ASTM D792 standard was followed to measure the densities of the parts using a Mettler Toledo MS303TS/00 density kit. A JEOL JSM 6390 scanning electron microscope (SEM) under an acceleration voltage of 5 kV was used to observe the morphologies at the cross sections of printed foams. Prior to SEM, samples were cryofractured and Au sputter coated using a Denton vacuum sputter coater for 6 min at currents between 3 and 4 mA.

#### 2.3.2 Flexural Testing

**Figure 1:**
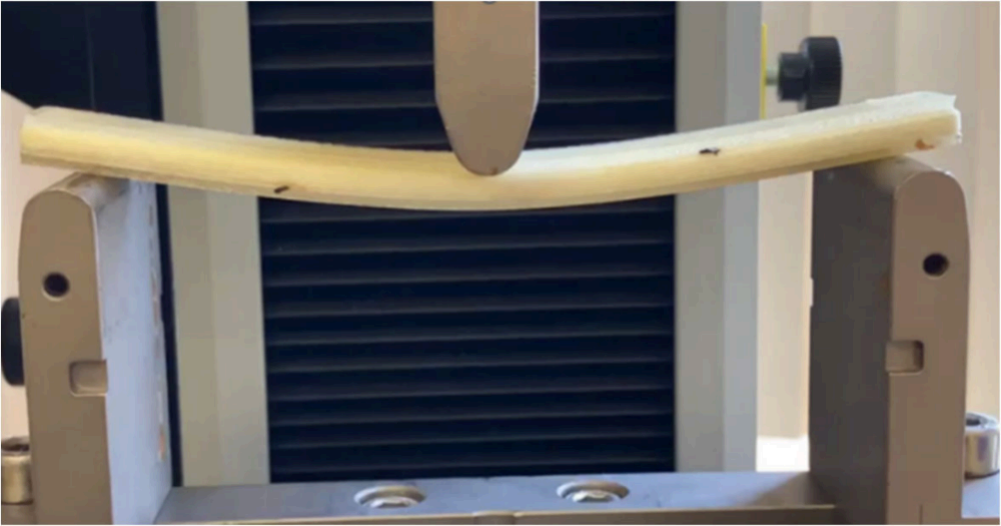
Three-point bend testing in action on an FGF flexural sample.

The ASTM D790 standard was followed to investigate the flexural response of the SDF, FGF, and solid unfoamed PLA flexural samples. Figure 1 shows the three-point flexural testing setup with a support span length *L* of 115 mm. Flexural samples had a total thickness, *H*, of 7.2 mm, width, *w*, of 15 mm, and length, *l*, of 138 mm. The rate of the crosshead motion in mm min^–1^ was calculated using eq 1, where *Z* is the strain rate set at 0.01 (mm mm^–1^) min^–1^, *L* is the support span length (mm), and *H* is total thickness of the sample (mm).
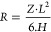
1

Testing was conducted on an Instron machine with a load cell capacity of 2 kN. At least five replications of flexural samples were tested for each case, and the mean and standard deviations are reported.

## 3 Results and Discussion

### 3.1 Filament Fabrication

For the ePLA filament where the loading level of TEM is 8.0 wt %, the objective was to fabricate the unexpanded filaments without letting TEM particles expand during the extrusion process. Barrel zone temperatures of Z1, Z2, Z3, Z4, and Z5 were set at 145, 151, 151, 147, and 125 °C, respectively, with a screw speed of 4 rpm. Z1 denotes the pellet feeding zone; Z2 and Z3 denote the melt compression zone; Z4 denotes the metering zone of the screw; and Z5 is the zone just before the die. Barrel zone temperatures (Z1–Z4) were successfully lowered enough (i.e., below the *T*_start_ temperatures (160–180 °C) of TEM) so as to provide good balance between the torque for mixing and simultaneously suppressing the foaming. The Z5 temperature was set lower at 125 °C to increase the viscosity of the extrudate and achieve optimum melt strength for a controlled filament diameter. The measured die melt temperature and die pressure were 145 ± 2 °C and 6.5 ± 0.3 MPa, respectively.

**Figure 2:**
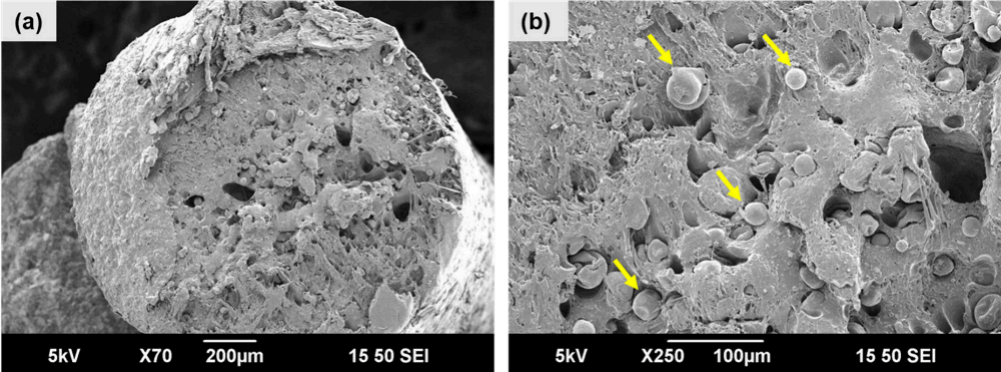
SEM micrographs of (a) ePLA (PLA/TEM8.0 wt %) filament cross-section and (b) magnified view revealing the distribution and size of TEMs (yellow arrows).

For a neat PLA filament, as there was no control needed to suppress the foaming during the filament extrusion process, the barrel zone temperatures of Z1, Z2, Z3, Z4, and Z5 were set relatively higher at 150, 202, 199, 170, and 145 °C, respectively, with a screw speed of 8 rpm. Die melt temperature and die pressure were recorded to be 183 ± 2 °C and 11.7 ± 0.3 MPa, respectively. Both extruded ePLA and neat PLA filaments were passed through a water bath and collected using a filabot spooler at a controlled diameter of 1.65 ± 0.03 mm. As seen in Figure 2, the TEM particles are well dispersed in the PLA matrix and the majority of TEMs are at their unexpanded state (less than 30 μm, as reported by the manufacturer), representing a good control over the foam suppression during the filament extrusion process.^25,40^ It is also noted that some air pockets (Figure 2) were observed, which could be due to the excessively low processing temperatures. The measured densities of ePLA and the neat PLA filament were 1.12 ± 0.02 g cm^–3^ and 1.22 ± 0.01 g cm^–3^. The reported densities of PLA, PE, and TEM are 1.24, 0.97, and 1.10 g cm^–3^, respectively. Based on the rule of mixtures with 8 wt % TEM and 8 wt % PE in a PLA matrix, the calculated density of ePLA is 1.19 g cm^–3^, which is slightly higher than the measured density. This difference could be attributed to the creation of some air pockets due to low-temperature extrusion and possibly some minor expansion of TEMs.

### 3.2 Co-extrusion Design, Setup, and Calibration

#### 3.2.1 Hot End with Static Mixer Nozzle

**Figure 3:**
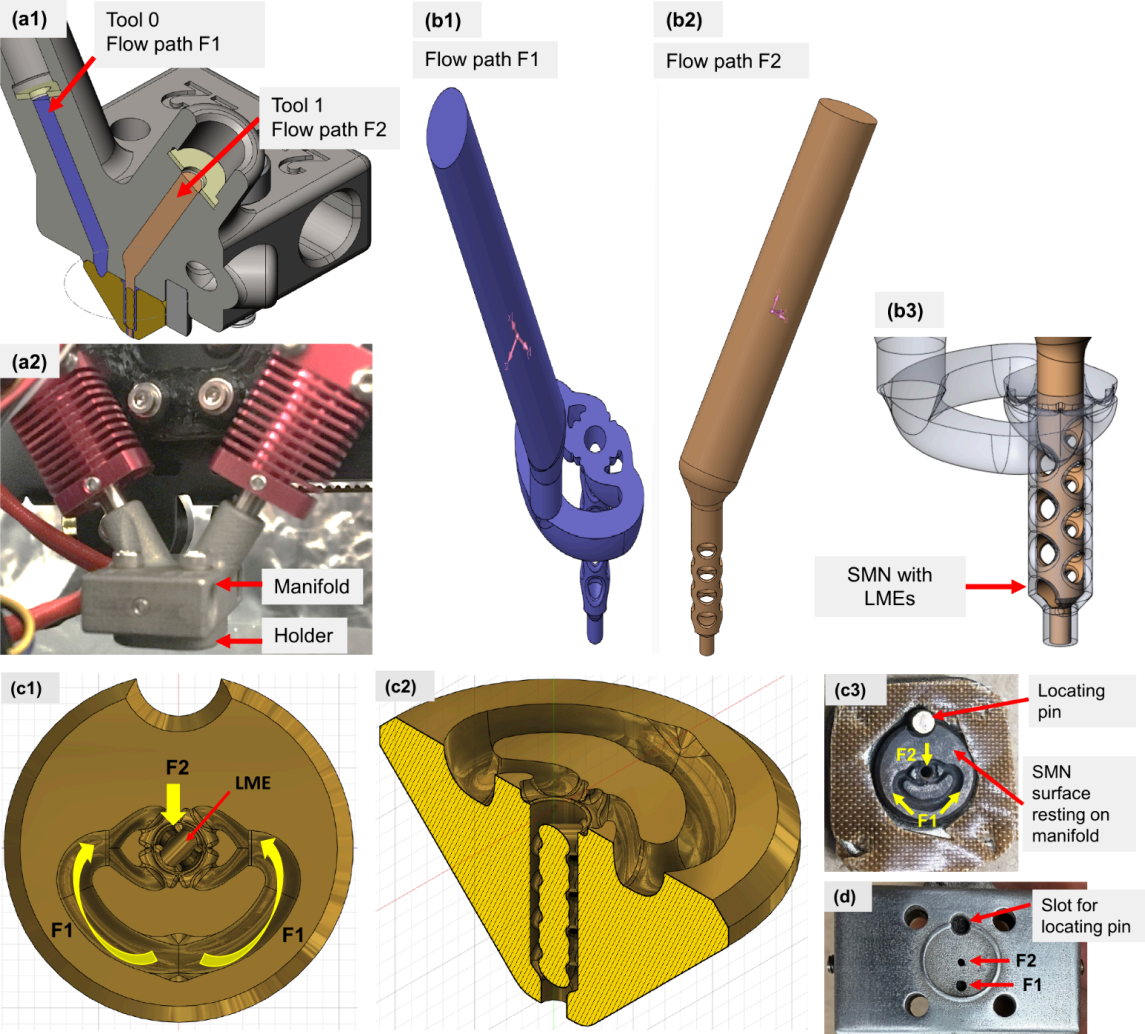
(a1) 3D CAD design showing the hot-end assembly, (a2) actual setup of the co-extrusion hot-end assembly, (b1) F1 flow path from tool 0, (b2) F2 flow path from tool 1, (b3) combined F1 and F2 flows inside the SMN where holes indicate the location of LMEs, (c1) top view of the SMN design where yellow arrows indicate F1 and F2 flow paths and the red arrow points to the top LME, (c2) 3D view of the SMN showing all eight LMEs sectioned, (c3) top view of the SMN showing its manifold rest area and the locator pin, and (d) the manifold which houses the heater and thermocouple and connects the SMN to the rest of the printer.

The density change in this approach relies on in situ control of the blowing agent amount (i.e., TEM content). Two filaments, i.e., ePLA and PLA, were fed into the hot-end assembly of the SMN. Both filaments were concurrently fed into the mixer with controlled feeding ratios, thereby changing the material composition of the foamed extrudate. Hence, by varying the material composition, the TEM content inside the in situ SMN was controlled from 0.0 wt % (i.e., 1.0 PLA feeding) to 8.0 wt % (i.e., 1.0 ePLA feeding), and thereby the degree of foam expansion and thus the density was varied. Figures 3(a1) and 3(a2) show the overall design and actual assembly of the hot end with the SMNs, where Tool 0 and Tool 1 are associated with flow path 1, F1, and flow path 2, F2, respectively. Figures 3(b1) and (b2) are the 3D representations of the F1 and F2 flow paths, and Figure 3(b3) depicts an enlarged 3D view of the SMN section where F1 and F2 merge and mix. The hollow regions in Figure 3(b3) denote the LMEs. Figure 3(c1,c2) show the top and sectioned isometric view of the SMN where F1, F2, and LMEs are labeled. Figure 3(c3) also gives the picture of the SMN from the top view identifying the flow paths and locator pin. F2 directly enters the mixer chamber from the top, right above the LME, but F1 enters the mixer chamber from the sides after passing through the curved channels. Finally, Figure 3(d) shows a photo of the manifold that houses the heater and thermocouple and connects the SMN to the rest of the printer. A locator pin is used to prevent the relative rotational motion of the SMN and the manifold. The flow channel connectors of F1 and F2 are also identified in Figure 3(d). The melt directly flows through the F2 opening of the manifold and enters the SMN. However, the melt passing through F1 needs to branch out in two flows circumferentially and enter the SMN from the sides (Figure 3c1 and c2). To reduce the flow resistance at the exit of F1 where the melt must turn and to better facilitate the flow, the diameter of F1 in the manifold inlet was designed slightly larger than that for F2. The protruded ring on the manifold provides sealing of the melt to prevent it from leaking out. Also, a Teflon film (as shown in Figure 3(c3)) was added at the interface to enhance the sealing between the SMN and the manifold.

Overall, flow path F1 provides a longer path and thus a greater residence time in the SMN, compared with that of F2. The volumes of F1 and F2 flow channels inside the SMN, calculated using the CAD design, were 16.42 and 2.58 mm^3^, respectively, accounting for a 13.84 mm^3^ larger volume for F1. For a filament volumetric feed-in flow rate of 2.76 mm^3^ s^–1^, corresponding to print conditions given in Table 3 for foamed samples, the residence time inside the SMN for F1 and F2 flow channels was calculated to be 5.93 and 0.93 s, respectively. Consequently, depending on whether ePLA passes through F1 or F2, the residence time will be different, which will affect the degree of TEM activation and expansion and thus the density.^26^ This is further discussed in Section 3.2.3. It is noted that the foamable filament (ePLA) residence time will also be affected by the mixing ratio of PLA and ePLA, which governs their absolute volumetric flow rates.

**Table 2:** Various Flow Path and Material Ratio Combinations for ePLA and PLA Filaments^a^

Sample no.	ePLA flow path	Bottom section	ePLA:PLA ratio in the middle section	Top section	SEM morphology
FGF1	F1	1.0 PLA	0.90:0.10	1.0 ePLA	Figure 6a
FGF2	F1	1.0 PLA	0.75:0.25	1.0 ePLA	Figure 6b
FGF3	F1	1.0 PLA	0.50:0.50	1.0 ePLA	Figure 6c
FGF4	F2	1.0 PLA	0.90:0.10	1.0 ePLA	Figure 7a
FGF5	F2	1.0 PLA	0.75:0.25	1.0 ePLA	Figure 7b
FGF6	F2	1.0 PLA	0.50:0.50	1.0 ePLA	Figure 7c

a F1 and F2 are flow paths 1 and 2, respectively.

**Table 3:** Fixed Print Process Parameters Utilized to Print SDF, FGF, and Neat PLA Flexural Samples

Fixed print process parameter	Value
Flow rate (%)	35
Layer height (mm)	0.2^a^, 0.4^b^
Nozzle temperature (°C)	200
Bed temperature (°C)	50
Mixer nozzle diameter (mm)	0.8
Raster width (mm)	0.8
Print speed (mm s^–1^)	25
Infill pattern	lines at 0°

a For PLA solid sections.

b For ePLA containing foamed sections

As shown in Figure 3(c2), the SMN incorporated eight LMEs with the function to distribute and mix the two incoming melt streams of F1 and F2. The LMEs were incorporated in an alternating orthogonal manner, all equally spaced in the nozzle axial direction. At the nozzle exit of SMN, the nozzle diameter was 0.8 mm. LMEs^41^ have been used in polymer processing to create multilayered sheets,^42,43^ tubes,^44,45^ and simple hierarchical structures.^46^ LMEs operate by stretching a layered flow strip to a larger width, cutting the strip into two pieces across its width, and then positioning the two strips of flow on top of each other for subsequent processing. The LMEs of the current work were designed following the work of Kazmer et al.,^47^ who recently conducted flow simulation using the level set method^48^ to predict and optimize multiplying elements for architected composites. In the implemented design of Figure 3(b3), the flow encounters eight cutting elements that should provide highly distributive and dispersive mixing of the materials incoming from the F1 and F2 channels.

#### 3.2.2 Foam Printing Optimization with a Single Filament

The LMEs integrated in the SMN are likely to provide relatively higher shear-induced heat compared with the conventional nozzles with no such resistance. Apart from that, the utilized printer (Creality CR-X Pro) operates on a Bowden tube type extruder drive rather than a direct extruder drive. Hence for this custom printer setup, optimization of process parameters was required before any further trials. A base G-code file was first generated by using Cura slicing software to print tensile bar samples. Using ePLA as the feedstock filament through tool 0, SDFs were printed. After screening trials, the print process parameters were found to be a nozzle temperature of 200 °C, bed temperature of 50 °C, print speed of 25 mm s^–1^, layer height of 0.4 mm, and nozzle diameter of 0.8 mm. Nozzle temperature was kept constant at 200 °C, which is an approximate middle value for the temperature range from *T*_start_ (160–180 °C) to *T*_max_ (210–230 °C) for the utilized TEM particles, and also it is a commonly used temperature for PLA.^49,50^ A layer height of 0.4 mm was also used in the foamed sample (as opposed to 0.2 mm in solid cases) to accommodate the foam expansion during raster deposition.

**Figure 4:**
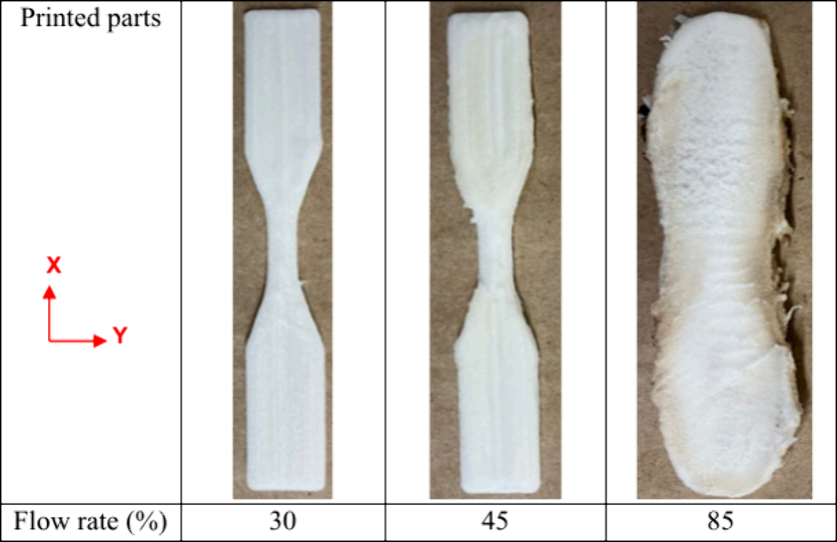
3D-printed single density foam parts in the XY plane at several flow rates.

With the print conditions above, single density foam samples were then printed at several flow rates to determine the optimal range of the flow rate for this custom setup. Figure 4 shows tensile bars printed at example flow rates of 85, 45, and 30% of the machine’s default 100% flow rate. A flow rate of 30% showed the best print quality with no under-extrusion nor over-extrusion. As the flow rate increased to 45%, the print started to exhibit minimal over-extrusion. Further increase to a high level of flow rate (85% in Figure 4) exhibited excessive foam expansion and thus resulted in severe over-extrusion. Samples printed with flow rates below 30% resulted in severe under-extrusion and were deemed unacceptable prints.

**Figure 5:**
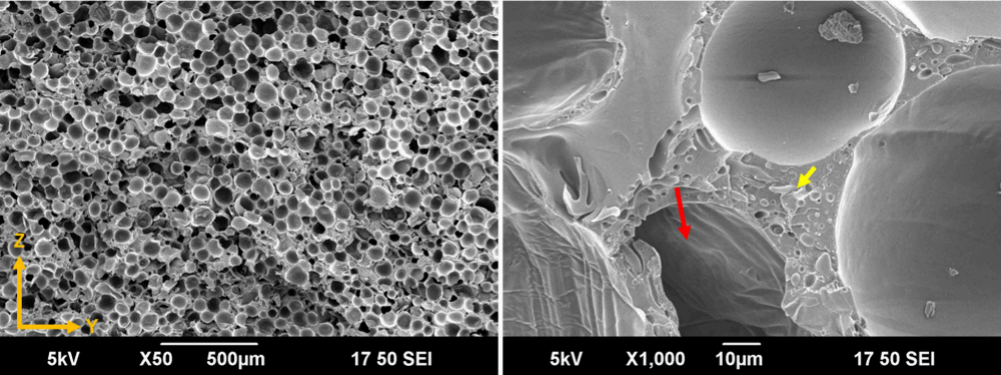
SEM micrographs of SDF printed parts at a flow rate of 30%. The images were taken in the ZY plane at different magnifications. The red arrow indicates the wrinkle effect on the expanded TEM’s shell material, and the yellow arrow indicates the PE fibrils from the TEM masterbatch.

Figure 5 shows the SEM images of the foams printed at a 30% flow rate. It is seen that the custom hot-end assembly was able to provide foams with uniform cellular morphology, and the presence of the LMEs did not negatively affect the overall cellular morphology. At higher magnification (1000×), some microspheres appeared to have wrinkle marks (red arrow in Figure 5) on their shell, which can be related to excessive gas loss, which could cause some shrinkage after expansion and result in a wrinkled shell. One potential reason for this could be the relatively low flow rate and thus long residence time inside the SMN. More details on the effect of the residence time on the cellular morphologies of the printed foams can be found elsewhere.^26^ However, overall, a very good cellular morphology was obtained, similar to those of previously reported printed foams.^24^ Some fibrils with a size of about one micrometer (yellow arrow in Figure 5) were also observed at high-magnification microscopy, which are polyethylene fibrils originating from the carrier polymer of the TEM masterbatch.^25^ One approach to avoid the wrinkling issue would be the utilization of a slightly lower nozzle temperature. Another way is to slightly increase the flow rate to lower the residence time. Considering the overall printability at 30% and 40% flow rate (Figure 4), the latter was adapted here and a flow rate of 35% was used for the rest of this study, while maintaining the other print process parameters unchanged. After the process optimization with a single ePLA filament, the next step was to print the FGFs with concurrent feeding and mixing of ePLA and PLA filaments.

It is noted that both nozzle temperature and flow rate have a strong influence on the foamability of filaments filled with TEM. The temperature regulates the thermal energy required for the TEMs to expand, and the flow rate controls the residence time given for the material to absorb the thermal energy and expand. As the focus here is to study the foamability by in line control of TEM content using the SMN and also noting that the effects of both temperature and flow rate have been extensively studied elsewhere,^26^ these two factors were kept unchanged in this work.

#### 3.2.3 Effect of Mixing Ratio and Flow Path on Foamability

**Figure 6:**
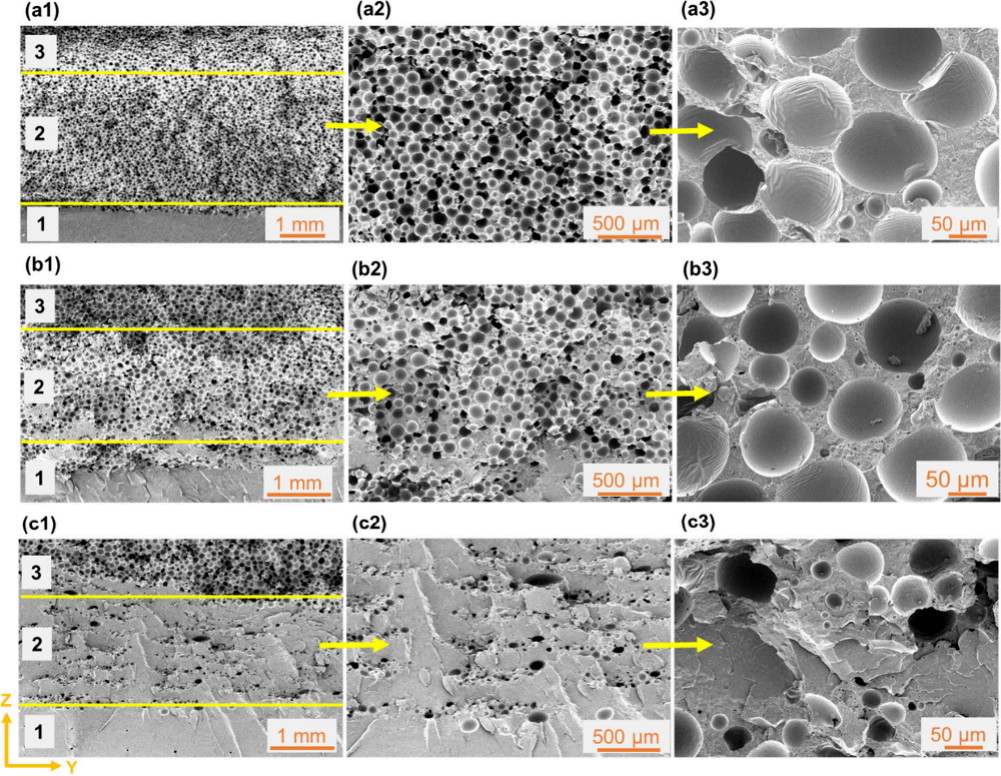
SEM micrographs depicting the overall cellular morphology of (a1–a3) FGF1, (b1–b3) FGF2, and (c1–c3) FGF3 samples (Table 2) in the ZY plane. In a1, b1, and c1, the bottom, middle, and top sections, identified as 1, 2, and 3, correspond to only PLA, an ePLA:PLA mixture, and only ePLA, respectively. Section 2 of FGF1, FGF2, and FGF3 samples had an ePLA:PLA ratio of 0.9:0.1, 0.75:0.25, and 0.5:0.5, respectively. The ePLA filament was passed through flow path F1 for all FGF1–3 samples.

**Figure 7:**
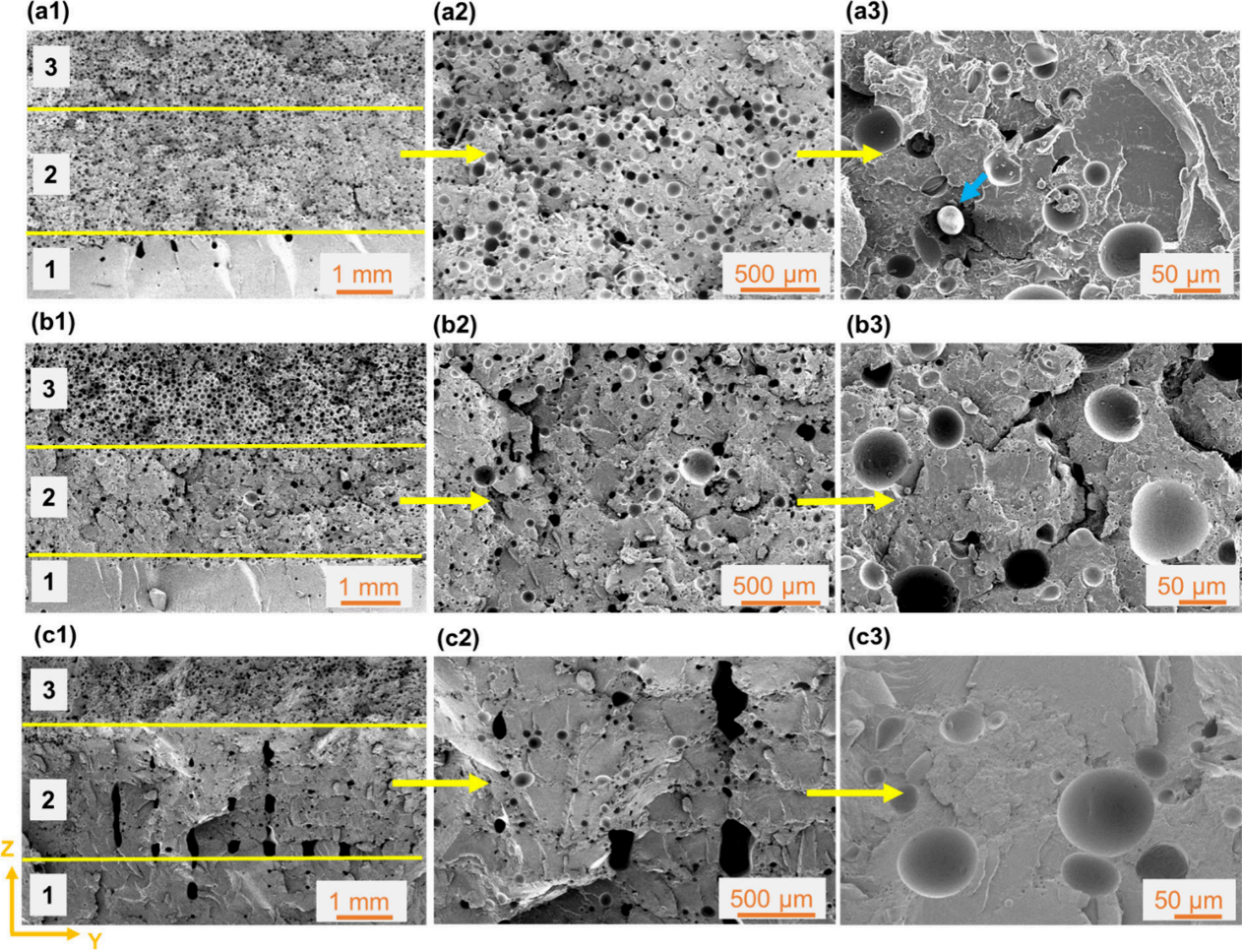
SEM micrographs depicting the overall cellular morphology of (a1–a3) FGF4, (b1–b3) FGF5, and (c1–c3) FGF6 samples (Table 2) in the ZY plane. In a1, b1, and c1, the bottom, middle, and top sections, identified as 1, 2, and 3, correspond to only PLA, the ePLA:PLA mixture, and only ePLA, respectively. Section 2 of FGF4, FGF5, and FGF6 samples had ePLA:PLA ratios of 0.9:0.1, 0.75:0.25, and 0.5:0.5, respectively. The ePLA filament was passed through flow path F2 for all FGF4–6 samples. In (a3) the blue arrow points to an unexpanded TEM particle.

The required modifications in the firmware and G-code files were made to enable concurrent dual filament feeding, as explained in Section 2.2. The FGF samples were designed with dimensions of *x* = 50 mm, *y* = 10 mm, and *z* = 8 mm, divided into three sections in the layer buildup (*z*) direction, and named section 1 (bottom), section 2 (middle), and section 3 (top) (Figures 6 and 7). The FGFs’ section 1 was printed with only a PLA filament, providing a solid unfoamed segment (e.g., section 1 in Figure 6(a1)), and section 3 was printed using only an ePLA filament, thus providing the maximum TEM content and the least density (e.g., section 3 in Figure 6(a1)). The middle section of FGFs was printed by mixing both filaments at ePLA:PLA ratios of 0.90:0.10, 0.75:0.25, and 0.50:0.50. This design enabled the variation of density from one section to another and also density variability in the middle section. The effect of flow path on the foaming behavior was also analyzed by switching ePLA and PLA filaments between tool 0 and tool 1 of the printer. Table 2 lists various FGFs, printed to study the influence of the mixing ratio and flow path of the two filaments on the foamability. Samples FGF1–FGF3 were printed with ePLA fed through flow path F1 (longer path), while samples FGF4–FGF6 were printed when ePLA was fed through flow path F2. Table 2 also identifies figures that depict the obtained cellular morphologies of each flow path/material ratio combination. Table 3 also shows the print process parameters used for all FGF1–6 samples.

Figures 6 and 7 depict the overall microstructures of samples FGF1–3 and FGF4–6 of Table 2, respectively. In both Figures 6 and 7, section 1 exhibits a solid unfoamed morphology, since it uses a 100% unfoamable solid PLA filament, whereas section 3 possesses a fully foamed cellular morphology, stemming from a 100% ePLA filament, which is foamable. Therefore, as expected, no significant differences were observed between Figures 6(a1), 6(b1), and 6(c1) in terms of their section 1 and section 3 microstructures.

Contrasting the micrographs of Figure 6 (FGF1–3) and Figure 7 (FGF4–6) reveals the effect of the ePLA flow path. The cellular morphologies obtained in section 2 of the FGF4–6 samples, which were made using flow path F2, were found to be significantly different, when compared to their counterpart FGF1–3 samples, made with flow path F1. Overall, smaller cell sizes and cell densities were obtained with flow path F2. The cell size and cell density values were significantly reduced to 51 μm and 3.2 × 10^5^ cells cm^–3^, respectively, in the 90% ePLA sample (compared to 76 μm and 11.3 × 10^5^ cells cm^–3^ of FGF1) and 47 μm and 1.7 × 10^5^ cells cm^–3^ in the 50% ePLA sample (compared to 68 μm and 9.5 × 10^5^ cells cm^–3^ of FGF2). Furthermore, for the 50% ePLA sample (Figure 7(c2)), interbead voids were relatively larger than those found in the FGF3 sample (Figure 6(c2)). The bigger interbead voids were created due to underfilling caused by insufficient expansion of TEM particles. In this case, the cell size and cell density values were estimated to be 46 μm and 0.8 × 10^5^ cells cm^–3^, respectively, which were the lowest among all the FGF samples. As explained in Section 3.2.1, the residence time of the molten polymer passing through flow path F2 was shorter than that in flow path F1. Hence, FGF4–6 samples had shorter times for the activation and expansion of TEMs, resulting in smaller cell sizes than those obtained for FGF1–3 counterparts. Traces of unexpanded TEM particles (marked by the blue arrow in Figure 7(a3)) were also observed, which is another indication that full foaming did not occur due to the lack of sufficient residence time inside the SMN hot-end assembly. Smaller cell densities in this case could be related to the unexpanded TEM particles. Another difference between F1 and F2 samples was the effect of flow rate. In the case of F1, with a decrease in the flow rate, the cell size decreased quite significantly, from 75 μm to 48 μm. However, in the case of F2, with a change in the ePLA flow rate, the cell size did not change that significantly and remained in the range of 46–51 μm. One potential reason for this could be that the F2 residence time was still short even at low flow rates. It can be concluded that the flow path design has a profound effect on the TEM activation and the degree of foam expansion. Adequate loading levels of TEM particles accompanied by sufficiently long melt flow yield uniform and homogeneous cellular morphologies with controllable cell size, cell density, and overall density, and it can be used to program FGFs.

### 3.3 Flexural Part Design, 3D Printing, and Testing

**Figure 8:**
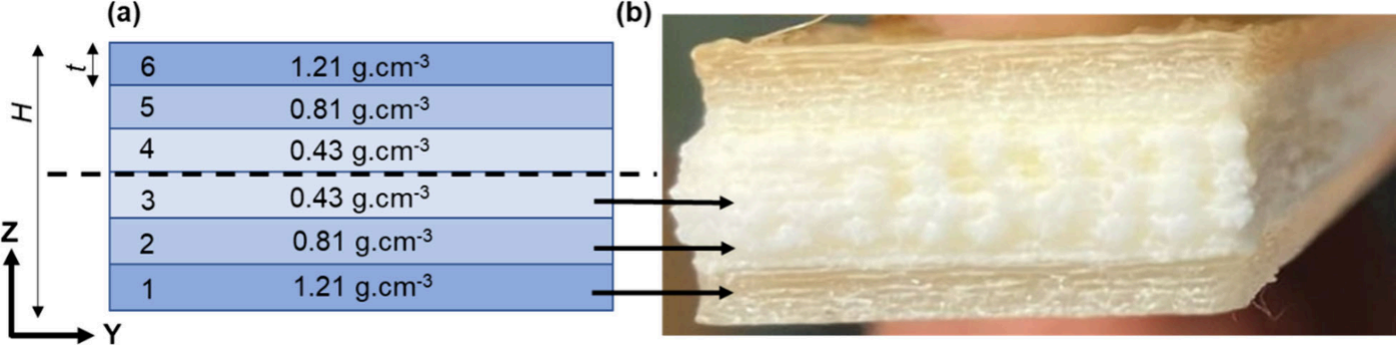
(a) Cross section of the designed FGF flexural sample in the ZY plane, where X is the print direction. The outer sections have the highest density, which decreases toward the core. *H* is the total height of 7.2 mm, and each section’s thickness, *t*, is 1.2 mm. (b) Side view of a 3D-printed FGF flexural sample showing sections having different densities.

After analyzing the effect of ePLA:PLA ratio and flow path on the cellular morphology, SDFs, FGFs, and solid PLA flexural samples were designed and printed for mechanical testing to assess the performance of the functionally graded beam. For the printing of FGF flexural samples, the ePLA filament was fed through flow path F1, whereas the PLA filament was fed through flow path F2. The same print process parameters were used as shown in Table 3 of Section 3.2.3. As shown in Figure 8(a), the FGF flexural samples were symmetrically designed with six sections divided equally with a section thickness, *t*, of 1.2 mm, total thickness, *H*, of 7.2 mm, and length, *l*, of 138 mm. Sections 1, 2, and 3 were printed with ePLA:PLA ratios of 0.0:1.0, 0.6:0.4, and 1.0:0.0, such that gradient densities of 1.21, 0.81, and 0.43 g cm^–3^, respectively, were obtained. These ratios were determined through initial screening experiments conducted at several different mixing ratios. Sections 4, 5, and 6 are mirror images of sections 3, 2, and 1, respectively. Figure 8(b) also shows the side view of a printed FGF flexural sample, where sections 1, 2, and 3 are identified by black arrows.

The SDF and solid PLA flexural samples were monolithic geometries with densities of 0.62 and 1.21 g cm^–3^, respectively. The printed flexural samples were found to have length (*l*) × width (*w*) × height (*H*) of 139.1 ± 0.31 × 16.1 ± 0.22 × 7.4 ± 0.09 mm^3^, 138.6 ± 0.22 × 16.3 ± 0.14 × 7.9 ± 0.02 mm^3^, and 137.2 ± 0.25 × 15.5 ± 0.03 × 7.4 ± 0.21 mm^3^ for FGFs, SDFs, and solid PLA samples, respectively, which are relatively close to their desired values as per the dimensions given in Section 2.3.2.

**Figure 9:**
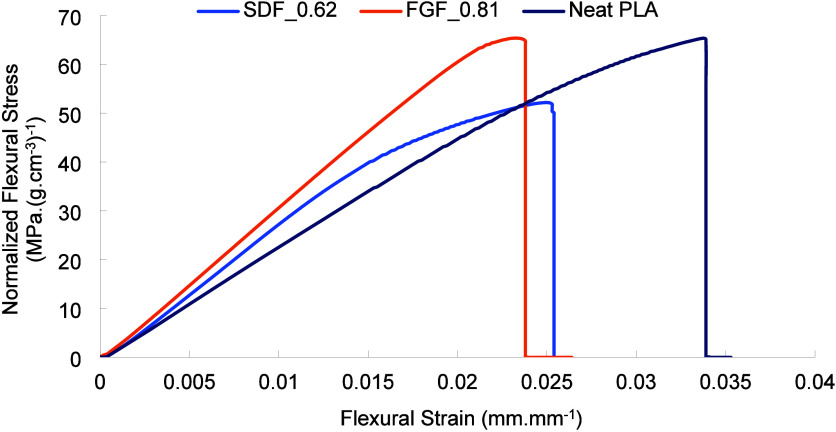
Representative normalized flexural stress–strain graph of single density foam (SDF_0.62) with a measured density of 0.62 g cm^–3^, functionally graded foam (FGF_0.81) with a measured density of 0.81 g cm^–3^, and solid PLA flexural samples with a measured density of 1.21 g cm^–3^.

Figure 9 shows the representative normalized flexural stress versus strain graphs of the single density foam sample (SDF_0.62) with a measured density of 0.62 g cm^–3^, the functionally graded foam sample (FGF_0.81) with a measured density of 0.81 g cm^–3^, and the solid PLA sample with a measured density of 1.21 g cm^–3^. Table 4 also tabulates the densities and flexural properties of all three types of flexural samples. The analytical density (*ρ*_analytical_) of FGF samples calculated based on the density of each section was 0.82 g cm^–3^, whereas the experimentally measured density (*ρ*_experimental_) was 0.81 g cm^–3^. This relatively small difference indicates the reproducibility and timely transition between the sections of variable densities during the printing process. For all the samples, the flexural test data were normalized with respect to their *ρ*_experimental_ values, and the normalized flexural modulus and normalized flexural yield strength are denoted as (*E*_f_)^n^ and (*σ*_f_)^n^, respectively. As seen in Figure 9 and Table 4, overall, FGF samples outperformed both SDF and solid PLA samples in terms of both normalized modulus and normalized yield strength. The solid PLA sample offered the least normalized modulus. The SDF sample exhibited the least normalized strength, which could be due to having 100% cellular structure. In particular, (*E*_f_)^n^ of the FGF sample was found to be 33% higher than (*E*_f_)^n^ of solid PLA samples and 15% higher than (*E*_f_)^n^ of SDF samples. In the case of strength, (*σ*_f_)^n^ of the FGF sample was 10% higher than (*σ*_f_)^n^ of solid PLA samples and 31% higher than (*σ*_f_)^n^ of SDF. Compared to SDF samples, higher values of (*E*_f_)^n^ and (*σ*_f_)^n^ for the FGF samples can be related to the higher stress resistance caused by the stiffer and stronger solid sections of PLA in sections 1 and 6 (Figure 8), while the remaining gradient sections with cellular structures (sections 2,3 and 4,5) effectively contributed to the density reduction. All samples had a relatively brittle failure after yielding, where solid PLA is the most brittle with a relatively smooth fracture surface.

**Table 4:** Density, *ρ*, Flexural Modulus, *E*_f_, Flexural Yield Strength, *σ*_f_, Normalized Flexural Modulus, (*E*_f_)^n^, and Normalized Flexural Yield Strength (*σ*_f_)^n^ of FGF, SDF, and Solid PLA Samples

Flexural sample	*ρ*_experimental_ (g cm^–3^)	*E*_f_ (MPa)	(*E*_f_)^n^ (MPa g_^-1^_ cm^3^)	*σ*_f_ (MPa)	(*σ*_f_)^n^ (MPa g_^-1^_ cm^3^)
FGF	0.81 ± 0.01	2588.41 ± 80.16	3195.57	52.47 ± 8.16	64.78
SDF	0.62 ± 0.01	1723.12 ± 36.38	2779.23	30.56 ± 1.05	49.29
Solid PLA	1.21 ± 0.01	2902.95 ± 48.71	2399.13	71.24 ± 6.51	58.88

Using classical composite beam theory^51^ or lamination theory^52^ with isotropic and homogeneous laminae, the effective flexural modulus, *E*_fE_, of the FGF laminate was also estimated.

To find the flexural modulus of each lamina, flexural samples having the density values of 1.21, 0.81, and 0.43 g cm^–3^, equivalent to those of sections 1, 2, and 3 (Figure 8), respectively, were printed and tested under the same conditions. The flexural modulus of each lamina, *E*_*i*_, was thus measured to be 2902.95 ± 48.71, 1432.68 ± 23.42, and 456.19 ± 17.3 MPa with densities of 1.21, 0.81, and 0.43 g cm^–3^, respectively. To calculate *E*_fE_, eq 2 was used:^51^
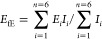
2where *n* = 6 is the number of laminae and *I_i_* is the second moment of area of lamina *i* about the neutral axis of the FGF sample. The *E*_fE_ was estimated to be 2430.29 MPa, accounting for only about 6% difference from the experimentally measured value of 2588.42, indicating that the composite theory can be applied in the design of such 3D-printed laminates with acceptable accuracy.

Overall, the flexural test results reveal the benefits of functionally graded beams against single density beams, whether solid or foamed. Using finite element analysis of sandwich beams with porous and graded cores, a previous report by Njim et al.^53^ has numerically demonstrated that the bending load increases with an increase in the gradient indices and decreases with an increase in the porosity factors. In another finite element study by Bonthu et al.,^54^ an increase in flexural strength of graded foams, compared to nongraded foams, was reported. Therefore, the trends observed here are in line with the computational predictions. The flexural test data confirm the capability of the proposed foam 3D printing method to fabricate functionally graded structure with enhanced performance through the dynamic control of foaming agent content in a simple extrusion additive manufacturing process.

## 4 Conclusion

In this study, we report the printing feasibility of functionally graded foams through in situ control of blowing agent content via a co-extrusion process equipped with a hot-end assembly of a static mixer nozzle. The SMN included two discrete flow paths and a mixer chamber, which was integrated with layer multiplying elements (LMEs). The hot-end assembly was first designed, made, and assembled. For the concurrent feeding of dual filaments, the printer’s firmware and G-code files were accordingly modified by adding mixing commands. A foamable ePLA filament (with 8.0 wt % of TEMs as blowing agent) and an unfoamable PLA filament were made using a single screw extrusion and then concurrently fed into the hot-end assembly of the SMN at controlled feeding rates. This provided various mixing ratios of ePLA and PLA. The effects of the ePLA:PLA mixing ratio and the flow path length on the foamability were thoroughly investigated. It was found that the ePLA:PLA ratio, varying from 90% to 50%, effectively controlled the cell size and cell density and, thus, dominated the overall foam density. Moreover, the longer flow path for ePLA proved to be more effective for foaming by providing a sufficient residence time for the expansion of the TEM particles. FGF flexural samples along with SDF and solid PLA samples were designed, printed, and mechanically tested. The results revealed that FGF samples outperformed both SDF and solid PLA in terms of the normalized flexural modulus and strength. For instance, 15% and 31% improvements in normalized modulus and strength were achieved by changing the design from SDF to FGF. This unique approach of the foam additive manufacturing process provides a facile and scalable method to design and print complex and functionally graded foams with programmable density profiles.

## Data Availability

Data are provided within the manuscript figures and tables.
